# Applications of machine learning techniques to predict filariasis using socio-economic factors

**DOI:** 10.1017/S0950268819001481

**Published:** 2019-09-02

**Authors:** Phani Krishna Kondeti, Kumar Ravi, Srinivasa Rao Mutheneni, Madhusudhan Rao Kadiri, Sriram Kumaraswamy, Ravi Vadlamani, Suryanaryana Murty Upadhyayula

**Affiliations:** 1 Bioinformatics Group, Department of Applied Biology, CSIR-Indian Institute of Chemical Technology, Hyderabad-500 007, Andhra Pradesh, India; 2 Centre for Excellence in Analytics, Institute for Development and Research in Banking Technology, Hyderabad-500 057, Telangana, India; 3 National Institute of Pharmaceutical Education and Research, Guwahati-781 032, Assam, India

**Keywords:** Filariasis, mosquito, socio-economic factors, Machine learning techniques

## Abstract

Filariasis is one of the major public health concerns in India. Approximately 600 million people spread across 250 districts of India are at risk of filariasis. To predict this disease, a pilot scale study was carried out in 30 villages of Karimnagar district of Telangana from 2004 to 2007 to collect epidemiological and socio-economic data. The collected data are analysed by employing various machine learning techniques such as Naïve Bayes (NB), logistic model tree, probabilistic neural network, J48 (C4.5), classification and regression tree, JRip and gradient boosting machine. The performances of these algorithms are reported using sensitivity, specificity, accuracy and area under ROC curve (AUC). Among all employed classification methods, NB yielded the best AUC of 64% and was equally statistically significant with the rest of the classifiers. Similarly, the J48 algorithm generated 23 decision rules that help in developing an early warning system to implement better prevention and control efforts in the management of filariasis.

## Introduction

Lymphatic filariasis (LF) is a major public health problem in 73 countries of Africa, Asia, Oceania and the Americas. LF affects nearly 68 million people. Furthermore, 1.1 billion people are living at the risk of infection [1, 2]. After malaria, filariasis is the second most common vector-borne disease [3]. Approximately 36 million people live with the disabling effects of LF, 17 million people with chronic lymphedema and another 19 million men with hydrocele [1]. The global programme to eliminate lymphatic filariasis (GPELF) was initiated in the year 2000 with the aim to eliminate LF as a public health problem by 2020, by interrupting the transmission of the parasite through an annual Mass Drug Administration (MDA) programme [3]. During the MDA programme, the populations at risk of filariasis are treated annually with a single dose of ivermectin and albendazole (IVM  +  ALB) in sub-Saharan Africa or with diethylcarbamazine and albendazole (DEC  +  ALB) in other regions for a minimum period of 5 years.

LF is a vector-borne parasitic disease mainly caused by the parasitic nematodes *Wuchereria bancrofti*, *Brugia malayi* and *Brugia timori*, and transmitted by the *Culex* or *Anopheles* host vectors. Filaria parasites damage the lymphatic system and cause debilitating swelling of the limbs, known as lymphedema or elephantiasis. Filariasis is prevalent in 18 states and the Union Territories of India. Approximately 600 million people reside with the risk of filariasis in 250 districts, contributing to over 40% of the global LF burden [4]. In recent years, India has made a significant progress towards the elimination of LF [5]. As per the recommendation of the World Health Organization, five rounds of MDA programme (65% of coverage) with the DEC  +  ALB drug combination was successfully administered in LF endemic districts [5].

The transmission of filariasis is influenced by environmental, socio-economic and demographic factors and lack of knowledge about the disease spectrum. LF and hydrocele severely affect the poor populations living in low socio-economic conditions. Acute and chronic cases of LF severely affect the economy output and increase poverty in a community [6]. Therefore, prediction of the onset of filariasis, well before its occurrence, significantly improves prevention success. Hence, in the present study, data mining/machine learning (ML) algorithms are employed to predict filariasis, based on epidemiological and socio-economic data.

Computational models are very popular in many disciplines and are extensively used for epidemiological data classification, disease diagnosis and prediction too. Data mining is an interdisciplinary area combining ML, intelligent information systems, database systems, statistics and operations research. Data mining helps in extracting knowledge from data that helps in intelligent decision making. It has been applied to a variety of problems in healthcare in order to improve decision making. Based on the foregoing discussion, our problem statement is defined in the next sub-section.

### Problem statement

LF is commonly known as elephantiasis and is a profoundly disfiguring disease. The chronic form of LF leads to social stigma and social exclusion, which adversely impacts the mental wellbeing of afflicted persons [7]. Studies indicate that 94% of the countries with the lowest Human Development Index (HDI) are endemic for LF [8]. Important risk factors for vector-borne diseases are ethnicity, occupation, education, awareness, living standards and the socio-economic status of a family [9, 10]. Based on the studies carried out in Philippines and Guyana, it has been observed that there is a strong association between endemicity of LF and socio-economic status [11]. However, in other countries where LF is endemic, very little research has been conducted in order to understand the role of socio-economic factors on filariasis. Hence, the present study focuses on understanding the role of socio-economic factors on LF and on predicting the occurrence of LF using various ML methods. In this connection, longitudinal studies are carried out to collect epidemiological and socio-economic data on LF from Karimnagar district of Telangana. ML tools such as Naïve Bayes (NB), J48, logistic model tree (LMT), probabilistic neural network (PNN), JRip and gradient boosting machine (GBM) were employed to predict LF.

### Contributions

The contributions of this study are as follows:

- We developed a dataset comprising of epidemiological and socio-economic data for 30 villages of Karimnagar district of Telangana (then in united Andhra Pradesh) from 2004 to 2007.
- We obtained important ‘if-then’ decision rules in order to develop an early warning system for the occurrence of LF.

### Literature review

ML has gained immense popularity and is being exploited in several disciplines including epidemiology and public health. Due to the increase in clinical datasets, researchers applied ML techniques extensively for the diagnosis and prediction of various disease outbreaks [12]. Dhamodharan [13] predicted liver disease using Bayesian classification through NB and functional tree algorithms. Vijayarani and Sudha [14] applied LMT, multilayer perception (MLP) and sequential minimal optimisation (SMO) algorithms to predict heart disease. Similarly, Dbritto *et al*. considered three different classification methods, namely NB, logistic regression and support vector machine (SVM) for predicting heart disease [15]. Adamker *et al*. [16] considered demographic and clinical data to predict hospitalisation and Shigellosis clinical diagnosis using logistic regression, SVM and neural networks. They reported *F*-measures of 97.4% and 96.1% for predicting hospitalisation and Shigellosis clinical diagnosis, respectively.

ML techniques are effectively used for the diagnosis and prediction of Ebola based on clinical data [17]. Han *et al*. applied ML on rodent species that carry zoonotic pathogens which cause infection to humans [18]. Various ML algorithms such as support vector regression, step-down linear regression, gradient boosted regression tree, negative binomial regression, least absolute shrinkage and selection operator linear regression model and generalised additive model were employed to forecast dengue incidence in China [19]. Furthermore, MLP, SVM and NB were employed to classify Parkinson's disease-afflicted patients and healthy persons [20, 21]. Patil [22] proposed a hybrid prediction model for the prediction of type-2 diabetes in patients. He employed a *K*-means algorithm to select relevant samples, which were further classified using the C4.5 algorithm, and he reported an accuracy of 92.38% [22].

Similarly, maximum entropy-based niche modelling technique [23] was applied to assess the potential distribution of LF in Africa in future climate change scenarios. Taking a cue from these studies, the present study focuses on predicting LF based on socio-economic conditions and to help implement better prevention and control measures to mitigate LF.

### Proposed approach

Predictive classification modelling learns a function from training data and aims to make few errors possible when tested with previous unseen data. A large number of classification algorithms were developed and used in a variety of medical applications [24, 25]. Medical data often suffer from imbalance issue that severely affects the classification results. To overcome this problem, a balancing method can be applied to the dataset. Popular balancing techniques include undersampling, oversampling and a hybrid of both. Undersampling refers to randomly removing samples from the majority class. Oversampling is the process of replicating minority class samples multiple times. The process of balancing data by performing undersampling and oversampling in tandem is called hybrid sampling [26]. The current study is based on the classification of filariasis dataset using NB, J48, LMT, PNN, JRip and GBM. These algorithms are employed using WEKA and NeuroShell 2.0. Feature subset selection was performed using *t*-statistic. The performance of these algorithms is reported in terms of sensitivity, specificity, accuracy and area under ROC curve (AUC).

## Methods

**Fig. 1.**
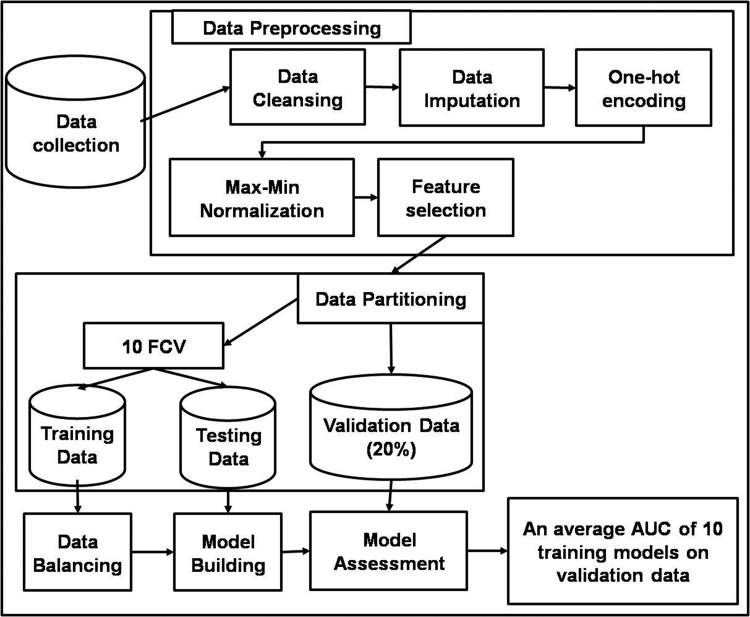
Schematic diagram of the proposed methodology.

The experiment was divided into six steps which involve data collection, data pre-processing, data partitioning, data balancing, model building and model assessment (Fig. 1). Pseudo code of the whole process is presented as Algorithm 1 (Supplementary information).

## Data collection

### Study area

The study was undertaken in 30 villages of Karimnagar district, Telangana (the then Andhra Pradesh) from 2004 to 2007. Karimnagar district lies 18°25′48″N, 79°9′90″E and is situated on the banks of river Manair, a tributary of the Godavari. The population of surveyed villages (30 villages) included either agriculturists or people engaged as labourers in the agricultural activity. The topography of the district is generally hilly and the altitude varies from 117 to 431 m between the villages where the study was undertaken. Karimnagar experiences dry climatic conditions with hot summers and cool winter. The southwest monsoon provides maximum rainfall to Karimnagar district.

### Data details

Epidemiological and socio-economic data were collected from the respondents/participants selected from all parts of the villages using a stratified random sampling methodology. The data were collected simultaneously by involving two sets of health volunteers. The socio-economic details were collected only from people who were involved in the epidemiological study.

### Epidemiological data

Using the finger prick method, 20 µl of the blood sample was collected from randomly selected 40 households per village (five persons from each household; 40 × 5 = 200 samples) between 20.00 and 23.00 h. During the epidemiological survey, 5394 blood smears were collected (Table 1) from 30 villages (approximately 200 samples from each village), stained with JSB-II (Jaswant-Singh-Bhattacherji) stain and then checked under a microscope for microfilaria (MF) [27].

**Table 1.** Epidemiological and socio-economic attributes for the prediction of filariasis

Major attributes	Sub-attributes	Survey participants (*n* = 5394)
Samples for filariasis	Samples positive for microfilaria	199
	Samples negative for microfilaria	5195
Age groups	1–5	173
	6–10	549
	11–17	1033
	18–25	831
	26–40	1430
	41–60	1209
	>61	169
Gender	Male	2623
	Female	2771
Occupation	Agriculture	2191
	Labourers	2135
	Business	586
	Employees	183
	Others	299
Education	Undergraduate	5067
	Graduate	327
House structure	Hut and thatched	1555
	Tiled	2372
	RCC	1467
Income (INR/Rs)	<1000	1251
	1000–3000	3380
	>3000	763
Breeding Habitats	Cess pool	991
	Cess pit	782
	Open drainage	1950
	No, breeding habitats	1671
Drainage system	Kutcha	1360
	Pucca	4034
Filaria awareness	Yes	3832
	No	1562
Participated in MDA programme	Yes	3144
	No	2250
Mosquito avoidance	Yes	982
	No	4412

### Socio-economic data

Socio-economic factors such as age, gender, use of mosquito avoidance measures (e.g. bed net, coils or no protection measures), awareness on filariasis, number of children in a family, place of residence, family's monthly income, house structure (living in a hut, thatched, tiled and reinforced cement concrete (RCC) structure), education details, occupation information, vector breeding habitats and participation in MDA programme have a possible influence on filariasis. The data were collected through interviewing the head of the family and other family members with a structured questionnaire (Table1).

Both epidemiological and socio-economic data were combined by using SQL query. From the combined table attributes, namely age, sex, house type, breeding habitat, drainage system, mosquito avoidance, awareness on filariasis, MDA and the target/class variable of filariasis were selected.

### Data pre-processing

The merged datasets based on the head of the family as a common factor were cleaned. We removed discrepancy in the data points such as extra spaces before or after categorical values, uses of mixed cases and unnecessary symbols. Subsequently, normalisation was performed to bring the data in the range of [0, 1] using max-min normalisation. The details of feature selection, data partitioning and data balancing are presented in the following sections.

### Feature selection

Feature selection is mainly used to select a subset of relevant features for building a robust learning model. Five feature selection methods namely gain ratio (GR), information gain, *χ*^2^, correlation and *t*-statistic-based feature selection methods were employed to select the most relevant features. Out of these feature selection methods, the GR feature selection method yielded the best performance. This method is briefly discussed here with the corresponding results.

### GR feature selection

GR is a non-symmetrical measure and is a different form of the information gain that reduces bias [28]. GR enhances information gain because it offers a normalised score of a feature's contribution to an optimal information gain, based on classification decision. GR is used in an iterative process where smaller sets of features are selected in an incremental fashion. These iterations terminate when a predefined number of features remain. GR is used because one of the disparity measures and high GR for the selected feature implies that the feature is highly discriminative and useful for further classification. GR was initially used in the decision tree (C4.5). It applies normalisation to information gain score by utilizing a split information value [29]. The GR of a feature (*A*) can be computed as follows:1
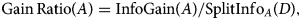
where InfoGain (*A*) is computed using the following formula:2
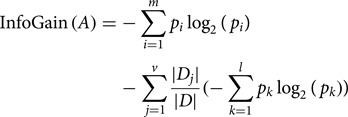
and Splitinfo_*A* 0_(*D*) is computed using the following formula:3
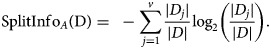


Here, 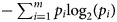 represents the entropy of an attribute *A* with respect to the whole dataset, {*D*_1_, *D*_2_, *D*_3_, …, *D*_*v*_} is the set of samples formed with respect to *v* distinct values of *A*, |*D*_*j*_| is the cardinality of *D*_*j*_, *|D|* is the cardinality of the whole dataset and 
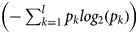
 represents the entropy of *A* with respect to *D*_*j*_ for the *l* number of classes available in *D*_*j*_ [30].

### Data partitioning and balancing

The whole dataset was divided into three parts for training, testing and validation, respectively. We separated 20% of the original data using stratified sampling as a validation test. The validation test was considered as unseen data for the models developed to report classification measures. The remaining 80% of the data were used to experiment under 10-Fold Cross Validation (10-FCV) framework. For the purpose of 10-FCV, we employed stratified sampling to maintain the ratio of positive and negative classes in the training as well as the test part identical to that of the original dataset. To improve the classification performance, different kinds of balancing techniques were employed on the training data in each fold. SMOTE and NON-SMOTE (oversampling, undersampling and hybrid sampling) techniques were applied for data balancing. The SMOTE technique was performed in R language [31, 32] and the non-SMOTE technique was performed through replication and removal of samples. In the oversampling method, the minority sample size was replicated to 100%, 200%, 300% and 400%, whereas 6% and 22% samples of majority classes were removed in the undersampling method. Here, the majority and minority classes belong to positive and negative classes, respectively.

### Model building

WEKA is a freely available open-source data mining tool implemented in Java. It consists of standard ML/data mining algorithms. Pre-processing and classification algorithms of WEKA generated insightful and useful knowledge from the filariasis dataset. Various classification algorithms such as NB, LMT, PNN, J48 (C4.5), classification and regression tree (CART) and JRip (repeated incremental pruning to produce error reduction (RIPPER)) were employed to predict filariasis. The techniques are briefly described below:

### Naïve Bayes

NB is an effective method for text classification [33]. It is a probabilistic classifier model based on the Bayes rule with an assumption of conditional independence of features [34]. It learns rapidly in various supervised classification problems.

Bayes' theorem explains the relationship between class variable ‘*y*’ and dependent features {*x*_1_, …, *x*_*n*_} as follows:4
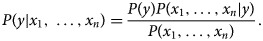


Using the naïve conditional independence assumption, the above equation can be written as5
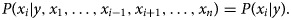


For all *i*, the given relationship can be simplified as6
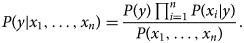


In the given equation, *P*(*x*_1_, …, *x*_*n*)_ is constant given the input. Hence, we can use the following classification rule:7
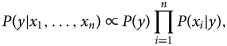
8
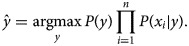


We use maximum *a posteriori* estimation to estimate *P*(*y*) and *P*(*x*_*i*_|*y*).

### Logistic model tree

LMT consists of a standard decision tree structure with logistic regression functions [35]. LMT starts to create a decision tree by fitting a simple linear regression function to the root node using the LogitBoost algorithm. This process is repeated for all child nodes in an iterative manner until the stopping criterion is met. After building the tree, pruning is performed using a CART algorithm.

### Probabilistic neural network

PNN is a powerful classification model. The advantages of PNN over a back-propagation network include faster learning and effectiveness on small datasets [36]. The architecture of PNN consists of four layers, namely input, pattern, summation and output layers. The input layer directly provides samples to the pattern layer which then calculates *Z* = *X.W*, where *X* is the input vector and *W* is the weight vector. Then, the pattern layer applies a Gaussian activation function on *Z*. The summation unit sums the probabilities of different categories into two groups for binary classification purposes. The output unit determines the class of a sample based on the ratio of *C*_*A*_ and *C*_*B*_. Here, *C*_*A*_ is the sum of a prior probability of class *A*, the loss function of class *A* and the number of samples in the class *B*. Similarly, *C*_*B*_ is the sum of a prior probability of class *B*, the loss function of class *B* and the number of samples in class *A*.

### J48 (C4.5)

J48 is a slightly modified form and an open source Java implementation of the C4.5 algorithm in WEKA [37]. C4.5 generates a classification-decision tree based on a given set of labelled input data by recursive partitioning of data. The decision tree is grown using a depth-first strategy. The algorithm considers all the possible tests that can split the dataset and selects a test that gives the best information gain. For each discrete attribute, one test with an outcome of as many as the number of distinct values of the attribute is considered. For each continuous attribute, binary tests involving every distinct value of the attribute are considered. To compute information gain for each binary test, the training dataset belonging to the node in consideration is sorted for the values of the continuous attribute. The information gain of the binary cut based on each distinct value is calculated in one scan of the sorted data. This process is repeated for each continuous attribute.

### Classification and regression tree

CART is one of the methods used to generate decision trees [38]. CART generates/induces either classification or regression trees, depending on the nature, i.e. categorical or numerical, of the target variable. CART performs binary splitting to grow a large tree on the training data. Gini index or entropy is considered to split the node, which is the measure of node purity. A smaller value indicates that the node contains samples belonging to the same class. Weka provides the implementation of simple CART algorithm, which stops growing tree when each terminal node has less than the pre-specified number of observations. The class of the test sample is decided based on the class of the majority of samples of the given terminal node. This algorithm is also suitable for datasets with missing values.

### JRip

JRip is a classification method in WEKA and is abbreviated as the RIPPER algorithm [39]. RIPPER algorithm can generate meaningful classification rules based on historical data. Classes are examined according to the increasing size and an initial set of rules for a class is generated using the incremental reduced error method. It proceeds by treating all the examples of a judgement in the training data as a class. In the next step, it finds a set of rules that covers all the members of that class. Thereafter, it proceeds to the next class and does the same, repeating this until all classes have been covered.

### Gradient boosting machine

GBM is an ensemble of weak prediction methods to perform classification and regression [39]. It performs bias-variance trade-off to maintain the balance between bias and variance. The loss function of GBM comprises a function to model errors. In GBM, many decision trees of small heights are fitted one after another without changing the existing trees in the model. Here, a decision tree is considered as a weak learner. A gradient descent method is employed to minimise the loss function when adding the trees. For every iteration, the parameters of the trees are estimated in such a way that each tree reduces the residual loss. This process is called functional gradient descent. In this way, the output of a new tree is added to the output of the existing sequence of trees to improve the final output of the model. GBM is prone to overfitting, which is prevented by regularisation.

## Model assessment criteria

The performance of all the seven models is assessed by using the following measures and are reported under the 10-FCV framework.9
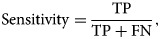
10
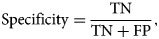
11
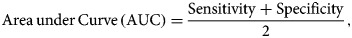
12
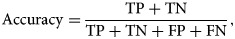
where TP, TN, FN and FP are the acronyms of true positive, true negative, false negative and false positive, respectively.

## Results

During the filariasis survey, blood samples of 5394 people were collected from 30 villages of Karimnagar district, of which 2771 (51.41%) were females and 2623 (48.68%) were males. Among the 5394 blood samples, 199 were found positive for MF (3.7%) and the rest of them were found to be negative for filariasis (Table 1). Both epidemiological and socio-economic data were merged based on the name of the head of the family. After merging, only 1041 records remained, comprising 890 (85.49%) of negative cases and 151 (14.15%) of positive cases. These 1041 records were pre-processed and normalised for model development. Seven classification models namely NB, J48, JRip, LMT, CART, GBM and PNN were applied to the unbalanced data without feature selection and the results are presented in Table 2.

**Table 2.** The results obtained for imbalanced dataset without feature selection

Classifiers	Specificity	Sensitivity	AUC	Accuracy	*t*-statistic for AUC
Naïve Bayes	0.92	0.11	0.51	0.80	1.8
J48	1.0	0	0.50	0.85	2.4
JRip	0.99	0	0.50	0.85	2.4
LMT	1.0	0	0.50	0.85	2.5
CART	1.0	0	0.50	0.85	2.4
PNN	0.57	0.33	0.45	0.36	3.35
GBM	0.62	0.53	0.57	0.60	–

Among all ML models, GBM yielded the best AUC with 57%. To test for statistical significance, *t* test was performed on AUC between GBM and each of the rest of the classifiers at 1% significance level and 18 degrees of freedom. Based on the *t* test, GBM outperformed PNN in a statistically significant way. However, the performances of the remaining classifiers are similar to that of GBM. The performance of GBM varies for each fold, because the model depends on various parameters such as the number of decision trees, the height of the tree and the number of samples per leave node. These parameters are changed for each fold. Hence, GBM is not statistically different from the other five classifiers.

Tables 3 and 4 indicate that feature selection methods and balancing techniques helped in increasing the classification performance. We performed oversampling of minority class by 100% (Table 3), 200%, 300% and 400% (Table 4) samples but that did not yield good results. We also performed hybrid sampling that comprises oversampling of minority class by 200% and undersampling of majority class by 22% but the results were, again, not satisfactory. The percentages of undersampling and oversampling depend on the dataset size and class proportions. PNN yielded the highest AUC and sensitivity compared to the rest of the classifiers and it statistically outperformed CART and JRip. Furthermore, PNN yielded statistically identical results to those of NB, J48, LMT and GBM. The study indicates that feature selection and balancing technique could not improve the performances of CART and JRip, whereas the performances of the rest of the classifiers improved using 100% oversampling. It also indicates that the minor improvement in the number of samples of minority class cannot affect the performance of CART and JRip.

**Table 3.** Results obtained using 100% oversampling and gain ratio feature selection

Classifiers	Specificity	Sensitivity	AUC	Accuracy	*t*-statistic for AUC
Naïve Bayes	0.83	0.29	0.56	0.75	2.02
J48	0.86	0.27	0.56	0.77	1.9
LMT	0.85	0.27	0.56	0.76	2.08
CART	0.94	0.06	0.50	0.82	4.78
JRip	0.94	0.06	0.50	0.81	4.57
PNN	0.80	0.47	0.63	0.75	–
GBM	0.63	0.58	0.61	0.63	0.65

**Table 4.** Results obtained using 400% oversampling and gain ratio feature selection

Classifiers	Specificity	Sensitivity	AUC	Accuracy	*t*-statistic for AUC
Naïve Bayes	0.60	0.68	0.64	0.61	–
J48	0.73	0.51	0.62	0.70	0.66
JRip	0.73	0.48	0.61	0.69	1.68
LMT	0.73	0.48	0.60	0.69	1.05
CART	0.73	0.44	0.58	0.69	1.08
PNN	0.53	0.64	0.58	0.54	2.15
GBM	0.65	0.61	0.63	0.64	1.28

**Fig. 2.**
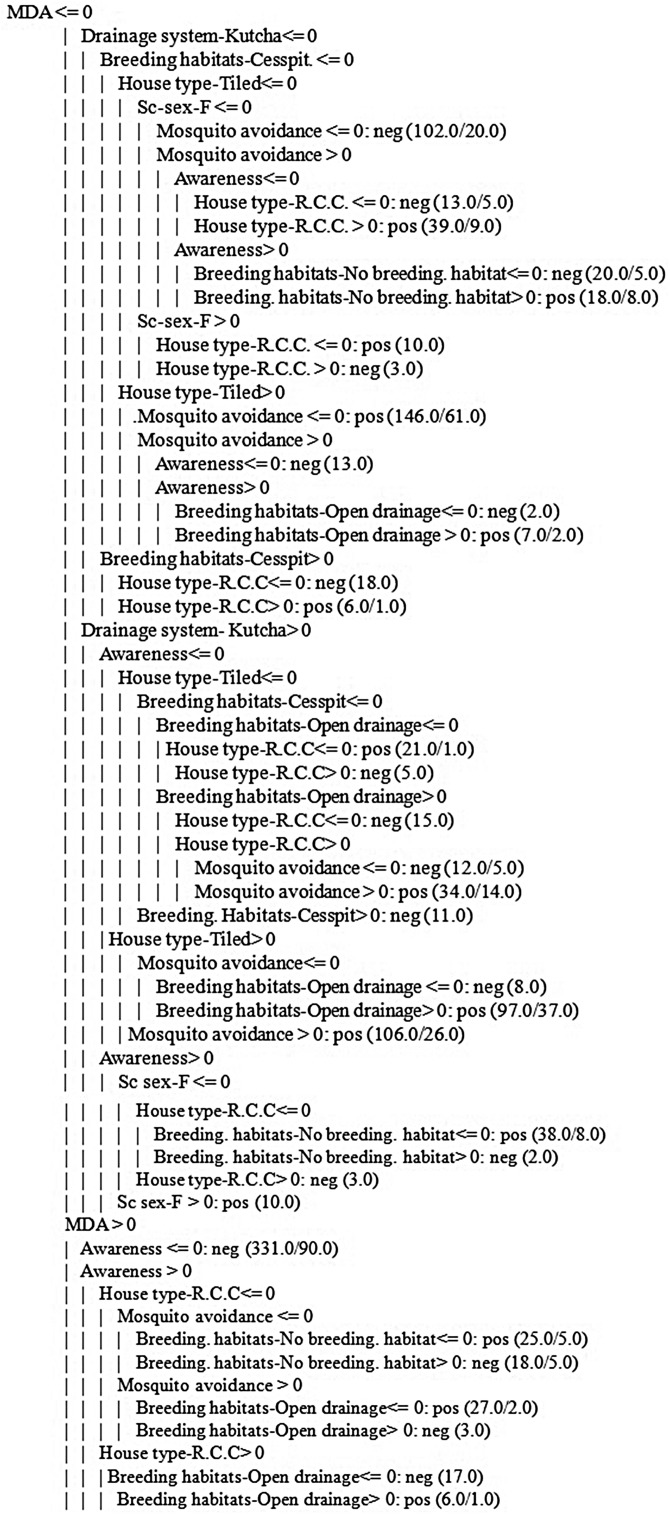
Tree generated using J48 algorithm.

**Fig. 3.**
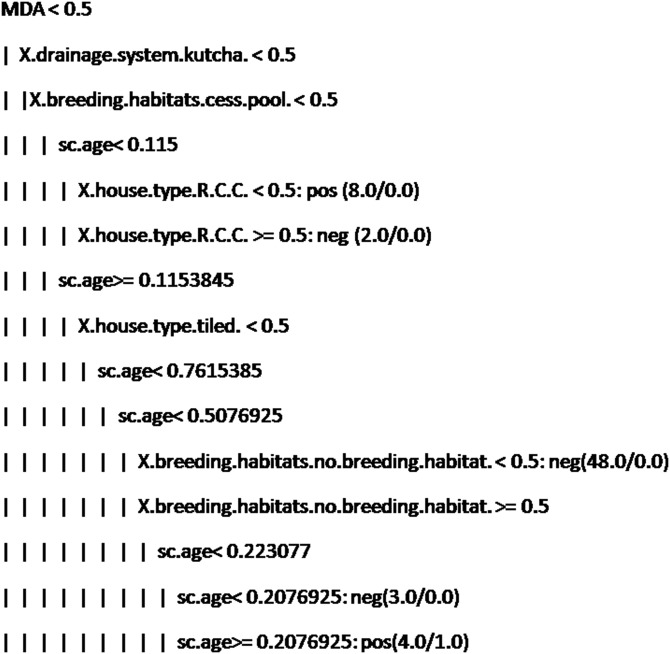
Decision rules obtained using CART.

Table 4 presents the results obtained using 400% oversampling with GR feature selection based on 12 features, namely *MDA, breeding habitats-open drainage and cesspit, no breeding habitats, kutcha drainage system, pucca drainage system, mosquito avoidance measures, tiled house, RCC house, awareness and gender*. NB yielded the best sensitivity and AUC compared to the other models. PNN yielded the second-best sensitivity. NB and PNN do not yield any rules, whereas J48 yielded classification rules and an AUC of 62% (Table 4). The number of rules generated by the J48 algorithm is presented in Figure 2. CART algorithm yielded the best result using default parameters with an AUC of 58% (Table 4). Default parameter set includes a minimal number of instances at the terminal node 2, cost-complexity pruning set to true and heuristics method for binary split set to true. The rules obtained by using CART are presented in Figure 3. The first two rules of Figure 3 can be interpreted as follows:

- If MDA is false and X.drainage.system.kutcha. is false and X.breeding.habitats.cess.pool. is false and sc.age < 11.74 years and X.house.type.R.C.C. is false, then the sample belongs to the positive class. Here, the number 8.0 in (8.0/0.0) indicates the number of training samples falling in positive class and no test sample belonged to the positive class.
- If MDA is false and X.drainage.system.kutcha. is false and X.breeding.habitats.cess.pool. is false and sc.age < 11.74 years and X.house.type.R.C.C. is true, then the sample belongs to negative class. Based on this rule, two training samples belonged to negative class and no test sample belonged to negative class.

To check for statistical significance, *t* test was performed between AUC obtained using NB and other classification models. It indicated that all methods are statistically similar.

**Fig. 4.**
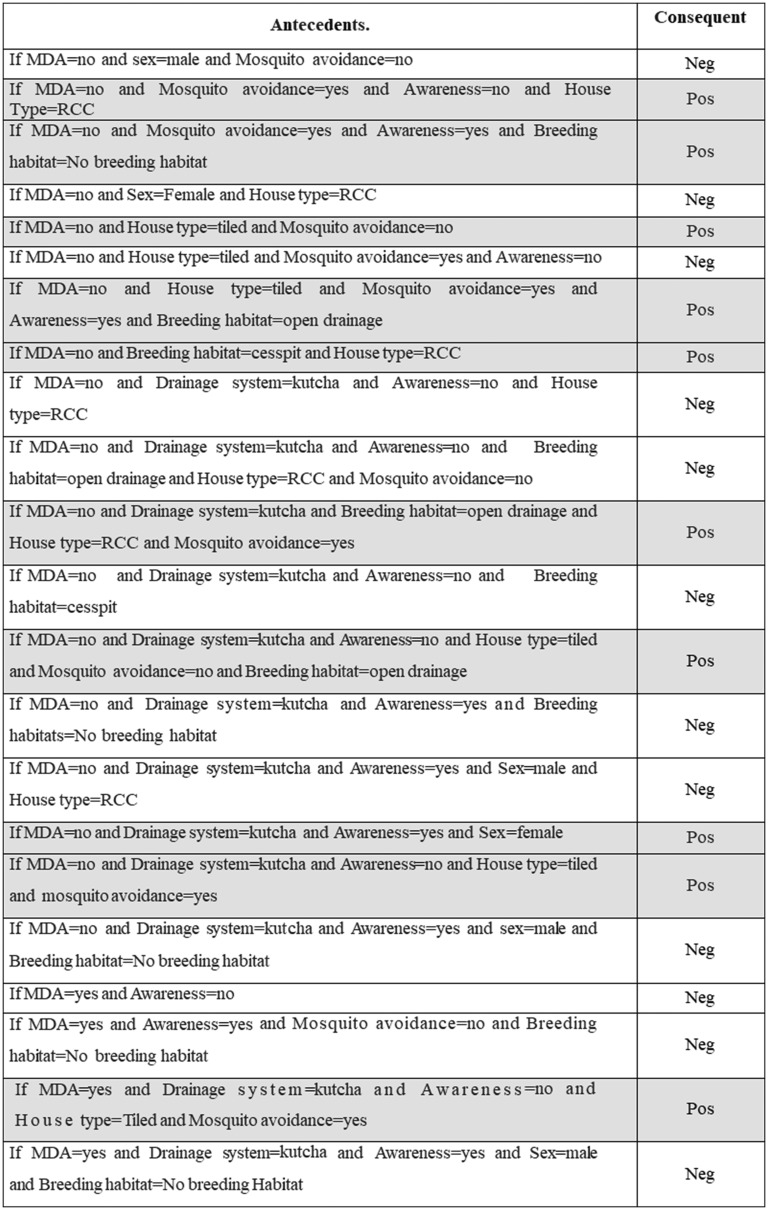
Rules generated by the J48 algorithm.

**Fig. 5.**
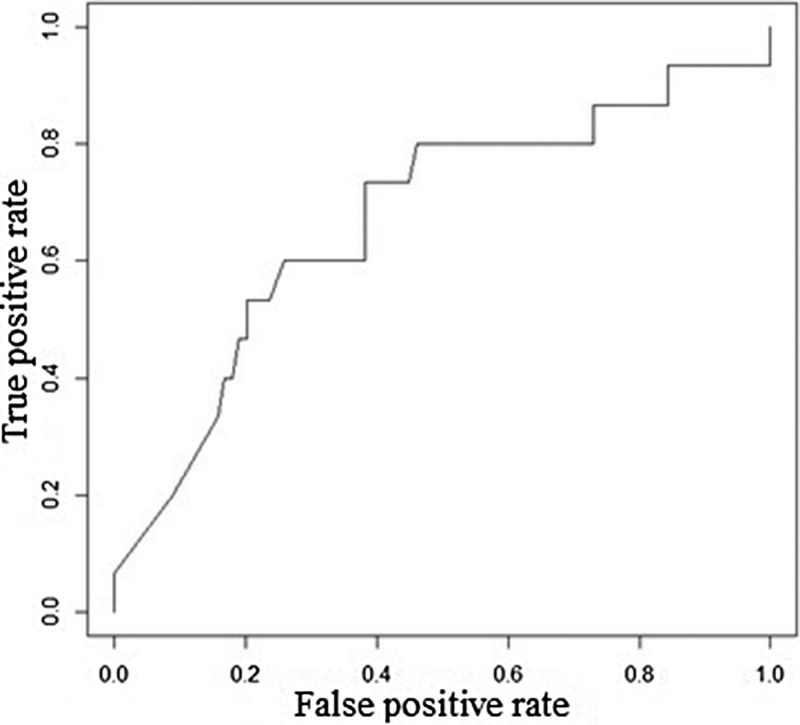
ROC area under the curve for GBM.

Table 5 explains the test sample dataset of filariasis based on the classification rules presented in Figure 4 and found that the ‘neg’ class was predicted accurately as ‘neg’ class by the model. In the present study, the filariasis data were subjected to classification by removing one feature at a time to test the feature capabilities. The sensitivity of the classifier decreased, which indicates the importance of the feature for filariasis prediction. After removing the feature, the model displayed a larger decrease in insensitivity when compared to the sensitivity of NB. This shows that the ‘*breeding habitats*’ feature has the highest impact on filariasis classification among all the features (Table 6). Similarly, relative feature importance obtained from Gini information and GBM shows that *MDA, mosquito avoidance, drainage system, awareness, house type, breeding habitats and gender* are important features (Table 7). ROC area under the curve is generated for the test data of onefold of 10-FCV and depicted in Figure 5.

**Table 5.** Test sample dataset

Variables	Test data
MDA	0
Breeding habitats – open drainage	1
Breeding habitats – no breeding habitat	0
Breeding habitats – cesspit	0
Drainage system – kutcha	0
Drainage system – pucca	1
Mosquito avoidance	1
House type – tiled	1
House type – RCC	0
Awareness	0
Sex – F	0
Sc sex – M	1
Remarks	Negative (neg)

**Table 6.** Results obtained using a different combination of variables

Feature	Specificity	Sensitivity	AUC	Accuracy	Impact
MDA	0.50	0.62	0.56	0.52	0.06
Mosquito avoidance	0.64	0.59	0.61	0.63	0.09
Drainage system	0.64	0.58	0.61	0.63	0.10
House type	0.66	0.50	0.58	0.64	0.17
Sex	0.65	0.49	0.57	0.63	0.18
Awareness	0.66	0.49	0.58	0.64	0.19
Breeding habitat	0.67	0.47	0.57	0.64	0.21

**Table 7.** Relative features importance obtained using GBM

Sl. No.	Feature name	Feature relative importance
1	MDA	17.8
2	Mosquito avoidance	11.3
3	Drainage system – kutcha	10.7
4	Awareness	9.91
5	Breeding habitats – open drainage	9.27
6	House type – RCC	8.26
7	Drainage system – pucca	8.12
8	House type – tiled	8.04
9	Breeding habitats – cesspit	7.22
10	Breeding habitats – no breeding habitat	6.23
11	Sc sex – M	1.6
12	Sex – F	1.56

## Discussion

LF is a major public health concern in India. Based on the present report, LF is widely spread in Karimnagar district of Telangana. The Government of India has launched the MDA programme since 2004 in endemic districts but, still, some sporadic cases are reported in the country. This may be due to many intrinsic factors such as lack of disease awareness, low socio-economic status, vector breeding habitats in and around the house and non-participation in mass drug programme [9]. Hence, the present study is focused on predicting the occurrence of filariasis using various ML algorithms. ML algorithms are used extensively in epidemiological and species distribution studies [40, 41]. ML algorithms help to understand the complex non-linear associations between the response (disease occurrence) and explanatory variables, and control for interactions among explanatory variables [42].

In the present study, we used various ML algorithms such as NB, J48, JRip, LMT, CART, PNN and GBM to predict filariasis with high accuracy. These classification algorithms produced more precise estimates and corroborated with different models to strengthen the robustness of the prediction. In terms of time complexity of classifiers, we found that NB has the least time complexity, because it determines the class of a sample simply based on posterior probability. Hence, the time complexity of NB is O(*n*), where ‘*n*’ is the number of samples, J48 and CART exhibit similar time complexity, but less than JRip, LMT, PNN and GBM. The time complexity of J48 and CART depends on the computation of split information, such as information gain and Gini ratio. The computation of split information depends on the number of distinct values that appeared in the number of features and time to prune the tree. As a result, the time complexity of J48 and CART was O(*mn*log*n*) + O(*n*(log*n*)^2^). JRip involves two stages, namely the building phase and the optimisation phase. Tree growing and pruning are performed in the building phase. Determination of an optimum number of rules is performed in the optimisation phase. These two processes are repeated multiple times by generating modified sets of rules at each iteration. Hence, the time complexity of JRip is O(*n*^2^) and PNN is represented as O(*n*). However, the space complexity of PNN is O(*n*) too, which is costlier than NB, J48, CART and JRip. The space complexity of PNN depends on the pattern layer, where it stores all the samples to decide the probability of class for the given sample. LMT and GBM are ensemble methods and these involve the use of multiple models to decide the class for the given sample. Hence, the time complexity of these classifiers is higher when compared to the rest of the classifiers.

LF is mainly transmitted by the Southern house mosquito *Culex quinquefasciatus*, the principal vector for filariasis in India. These vectors breed where there is a lack of basic sanitary conditions and prevalence of cesspools, cesspit and kutcha drains. These cesspools, cesspits and open drainage systems help to enhance the vector breeding habitats and increase vector density which leads to a higher risk of filariasis transmission, as observed [9]. Similarly, the lack of a proper drainage system in the study villages, and the presence of cesspits and cesspools are observed in and around the houses, which may favour the proliferation of *C. qninquefasciatus*. The ML models predict that, among all the socio-economic variables, the breeding habitats highly influence the occurrence of filariasis.

ML/data mining helps public health officials in decision making and real-time prediction of disease outbreaks. J48 yielded a set of classification rules which is considered as an early warning expert system for filariasis in Telangana, India. Policy makers should make appropriate plans to improve the socio-economic status of populations through better hygiene and sanitary conditions.

## Conclusions and future directions

We predicted the occurrence of filariasis based on socio-economic parameters using ML algorithms. NB yielded the best AUC (64%) using GR feature selection and 400% oversampling. Similarly, J48 yielded an AUC of 62% and yielded 23 classification rules based on six features, namely *MDA, gender, mosquito avoidance, house type, breeding habitat and drainage system*. From this study, it is observed that *gender, house type, breeding habitats, mosquito avoidance, drainage system, participation in MDA and awareness* directly influence the occurrence and spread of filariasis. Among all, the ‘*breeding habitats*’ feature has shown the highest specificity and impact on filariasis. Hence, the *breeding habitats* of mosquitoes need to be destroyed in low socio-economic zones to prevent further transmission of the parasite. The Government of India is moving towards the elimination of LF by 2020, but this study shows that filariasis is still thriving in communities even after the implementation of MDA programmes. Hence, more attention should be given to disease surveillance systems. Health awareness campaigns should be conducted to educate and increase the consumption of DEC in the target groups of endemic populations. The future direction of the study is based on the outcome of ML algorithms and an early warning system will be developed by adding some more important features such as immunological factors and climatic variables. The limitation of the study is that the method is not suitable for incremental learning if there are new data in the dataset. Therefore, in the future, incremental learning methods will be developed to address this limitation.

## Supplementary material

For supplementary material accompanying this paper visit https://doi.org/10.1017/S0950268819001481. click here to view supplementary material
