# Supplementary material for: Applications of machine learning techniques to predict filariasis using socio-economic factors
Source: Epidemiol Infect. 2019 Sep 2;147:e260. doi: 10.1017/S0950268819001481 (PMC6805759; doi:10.1017/S0950268819001481)
Supplement: Supplementary file 1 [file S0950268819001481sup001.docx]

**Algorithm 1: Algorithm of the whole process**

Step 1: Performing data cleaning like removal of extra spaces and standardization of the text.

Step 2: Performing data imputation to replace missing values.

Step 3: Performing one-hot encoding of the categorical variables.

Step 4: Performing max-min normalization to bring the numerical values in the range of [0,1].

Step 5: Performing feature selection with the help of domain expert and filter-based techniques.

Step 6: Data partitioning is performed to reserve 20% samples as validation set to report the final prediction measures.

Step 7: Performing data balancing using undersampling, oversampling and hybrid sampling techniques.

Step 8: Employing different machine learning algorithms to obtain the models.

Step 9: Fine tuning the model based on test samples.

Step 9: Reporting the AUC on validation samples i.e. unseen data.
